# Preservation of Cerebellar Afferent Pathway May Be Related to Good Hand Function in Patients with Stroke

**DOI:** 10.3390/life12070959

**Published:** 2022-06-26

**Authors:** Bo Kyung Shin, Hae-Yeon Park, Hanee Rim, Ji Yoon Jung, Sungwoo Paek, Yeun Jie Yoo, Mi-Jeong Yoon, Bo Young Hong, Seong Hoon Lim

**Affiliations:** 1 Department of Rehabilitation Medicine, St. Vincent’s Hospital, College of Medicine, The Catholic University of Korea, Seoul 06591, Korea; bogyeong0412@gmail.com (B.K.S.); camnd140@naver.com (H.R.); jennylidada@gmail.com (J.Y.J.); ys97157@naver.com (S.P.); nugry@naver.com (Y.J.Y.); allogen@naver.com (M.-J.Y.); byhong@songeui.ac.kr (B.Y.H.); 2 Department of Rehabilitation Medicine, Seoul St. Mary’s Hospital, College of Medicine, The Catholic University of Korea, Seoul 06591, Korea; hy2park@naver.com

**Keywords:** stroke, recovery, hand function, corticospinal tract, cortico-ponto-cerebellar tract, white matter, diffusion tensor imaging, DTI

## Abstract

Many chronic stroke patients suffer from worsened hand function, and functional recovery of the hand does not occur well after six months of stroke. Therefore, predicting final hand function after stroke through acute phase imaging would be an important issue in counseling with the patients or their family. Thus, we investigated the remaining white matter integrity in the corticospinal tract (CST) and cortico-ponto-cerebellar tract (CPCT) at the acute stage of stroke and chronic hand function after stroke, and present the cut-off value of fiber number (FN) and fractional anisotropy (FA) of CST and CPCT at the acute stage for predicting final hand function after the recovery period. This retrospective case-control study included 18 stroke patients who were classified into two groups: poor hand function with stroke (*n* = 11) and good hand function with stroke (*n* = 7). DTI was done within two months ± 15 days after onset, and the Jebson’s Hand Function test was conducted 6–12 months after onset. The investigation of white matter was focused on the values of FN and FA for CST and CPCT, which were measured separately. The normalized (affected/non-affected) FA and FN values in the CPCT in the good hand function group were higher than those in the poor hand function group. The normalized FN and FA values in the CST were not significantly different between the poor hand function group and the good hand function group. The normalized cut-off value that distinguished the good hand function group from the poor hand function group was 0.8889 for FA in the CPCT. The integrity of the CPCT in the acute stage was associated with hand function in the chronic stage after a stroke. Ultimately, the integrity of the CPCT in the early stage after onset can be used to predict chronic hand function. Based on these results, cerebellar afferent fiber measurements may be a useful addition to predict hand function and plan specific rehabilitation strategies in stroke patients.

## 1. Introduction

Impairment of hand function is a significant hurdle for restoring functional independence in stroke survivors. Many stroke patients suffer from upper extremity functional limitations. Previous studies have shown that upper extremity function is restored in less than 12% of patients six months after a stroke, even when they receive intensive rehabilitation [1,2]. Previous studies have focused on anatomical structures related to motor function, especially hand function, after a stroke. Several lesions, such as the internal capsule or injury of corticospinal tract (CST), have been found to be related to decreased hand motor function at chronic stage after stroke [3,4,5]. Another study showed that partially disrupted CST may benefit most from rehabilitation therapy for hand function recovery after hypertensive intracerebral hemorrhage [6]. In another view of function, after the stroke, the integrity of CST may be a predictive value for gait and the activities of daily living, as suggested by several reports [7,8]. In contrast to these results, our recent research showed that the integrity of CST might not be a predictive value for gait or balance [9]. Taken together, the CST may be a predictive value for several functions and be related to motor outcomes in patients with stroke. We demonstrated previously that the integrity of CST was related to the hand function at the chronic stage of stroke [3]. Recently, we demonstrated that the cortico-ponto-cerebellar tract (CPCT) may affect the hand function in normal subjects and in stroke patients [10,11].

We hypothesized that integrity of CST or CPCT in the acute phase could predict hand function in the chronic phase in patients with stroke. Thus, we investigated the relationship between the integrity of white matter, which are CST and CPCT and chronic hand function. In addition, we attempted to present a cut-off value for predicting chronic hand function through the DTI value at the acute stroke stage.

## 2. Materials and Methods

### 2.1. Study Design and Participants

This was a retrospective longitudinal observational study. We enrolled subjects that met the following criteria: (1) first incidence of a unilateral stroke, (2) 3T-magnetic resonance imaging (MRI) scan and brain DTI achieved within two months (two months ± 15 days) after onset; (3) evaluated by Jebson’s Hand Function test after six months after onset (6–12 months); and excluded (1) recurrent stroke; (2) after-stroke brain complications, such as hydrocephalus; (3) inflammatory arthritis or inflammatory myopathy; and (4) an underlying degenerative brain disease, such as Parkinson’s disease. Finally, eighteen subjects were enrolled and divided into two groups: poor hand function with stroke (*n* = 11) and good hand function with stroke (*n* = 7).

Hand function was evaluated by the Jebson’s Hand Function test. The Jebson’s Hand Function test consists of seven categories: writing; turning over 3 × 5 in cards; picking up small common objects; simulated feeding; stacking checkers; picking up bulky, light objects; and picking up large, heavy objects [12,13]. We recorded the time it takes for each of the seven items of the Jebson’s hand function test. When each item could not be performed even though sufficient time was provided, each item was marked as ‘uncheckable’. We classified the ‘good hand function group’ if none of the items were ‘uncheckable’, which means performing all items no matter how long it took. And the ‘poor hand function group’ indicates if any one of the seven items was ‘uncheckable’. All subjects received routine physical and occupational therapy for one to two h per day, five days per week. They also received speech therapy if needed. When the patients were fully recovered, rehabilitation therapy was stopped at a time determined at a team conference that included a physiatrist, therapists, and the patients themselves. The rehabilitation program for all subjects began within seven days after onset and continued until 6–12 months after onset [14,15].

Because the present study was a cross-sectional observational study investigating the relationship between DTI and hand function, the sample size was not planned before the study.

### 2.2. DTI Acquisition and Image Processing

DTI was performed with a 3.0 T magnetic resonance imager (MAGNETOM^®^ Verio; Siemens, Erlangen, Germany) equipped with a six-channel head coil. Data were received in the form of single-shot spin-echo echo-planar images, with axial slices covering the whole brain across 76 interleaved slices 2.0 mm in thickness (no gap; repetition time/echo time = 14,300/84 ms; field of view = 224 × 224 mm^2^; matrix 224 × 224; voxel size 1 × 1 × 2 mm^3^ (isotropic); number of excitations = 1). Diffusion sensitizing gradients were used in 64 noncollinear directions with a b-value of 1000 ms/mm^2^. The b = 0 images were scanned before obtaining the diffusion-weighted images, with 65 volumes in total [3,11,16].

Fiber tracking was based on the fiber assignment continuous tracking (FACT) algorithm and a multiple regions of interest (ROIs) approach using DTI-studio [3,16]. Pixel-wise outlier detection (i.e., Yue’s method) is used to compensate artifacts like eddy current, head motion, and cardiac artifact in DTI-studio [17,18]

### 2.3. Diffusion Tensor Tractography

**Figure 1:**
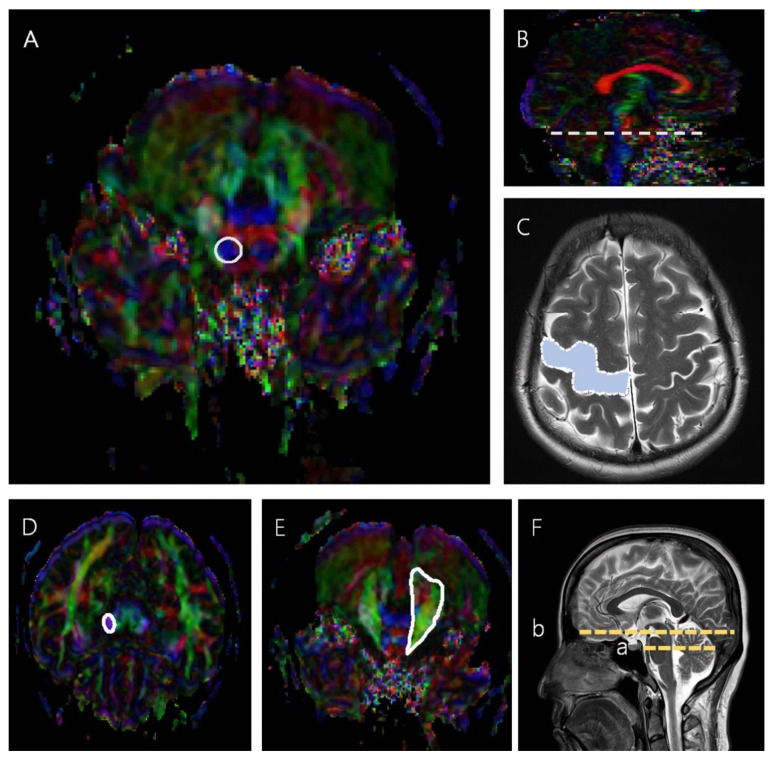
The seed and target ROIs used to reconstruct the CST and CPCT. (**A**) The region inside the solid line represents the seed ROI in the CST, mid-pons, in an axial color map. (**B**) The dotted line represents the mid-pons level in a sagittal color map. (**C**) The blue region in the dotted line represents the target ROI in the CST in an axial brain MRI scan. (**D**) The region in the solid line represents the target ROI in the CPCT at the cerebral peduncle in an axial color map. (**E**) The region in the solid line represents the seed ROI in the CPCT at the middle cerebellar peduncle, mid pons, in an axial color map. (**F**) (a) is the slice level of the seed ROI, and (b) is the slice level of the target ROI in a sagittal brain MRI scan.

The CST was reconstructed using two ROIs in which the seed ROI was placed on the mid-pons portion of the CST in the axial plane, and the target ROI was the M1 [4,19]. When reconstructing the CPCT, the seed ROI was placed on the middle cerebellar peduncle, and the target ROI was placed on the cerebral peduncle of the contralateral side (Figure 1) [10,20].

### 2.4. Statistical Analysis

The fiber number (FN) and FA values for the CST and CPCT were normalized as data for the affected hand/data for non-affected hand regardless of handedness [21]. The Mann-Whitney U test was carried out to assess differences between the two groups. The test was two-tailed and *p* values < 0.05 were regarded as significant. Receiver operating characteristic (ROC) curve analysis with FN and FA values of CST and CPCT was used to obtain the most useful cut-off value for predicting good hand function. Among the variables with an asymptotic significance < 0.05, the variable with the largest area under the curve (AUC) was selected and the sensitivity and specificity of each value were reviewed to obtain the cut-off value. All statistical analyses were executed using SPSS software for Windows (ver. 28.0; IBM Corp., Armonk, NY, USA).

## 3. Results

The demographic and clinical characteristics of the two groups are shown in Table 1. The distributions of age, sex, stroke type, brain injury location, and hemispheric lesion were not significantly different between the two groups. The FN and FA values in the CST and CPCT for the two groups are shown in Table 2.

**Figure 2:**
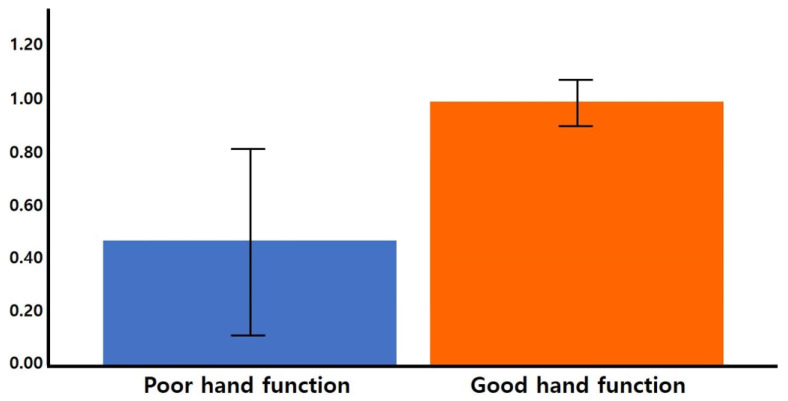
The normalized (affected/non-affected) FA in the CPCT. The mean values with CIs are shown as bars. The FA value of the CPCT in the poor hand function group was lower than in the good hand function group (*p =* 0.015). CI, confidence interval.

**Figure 3:**
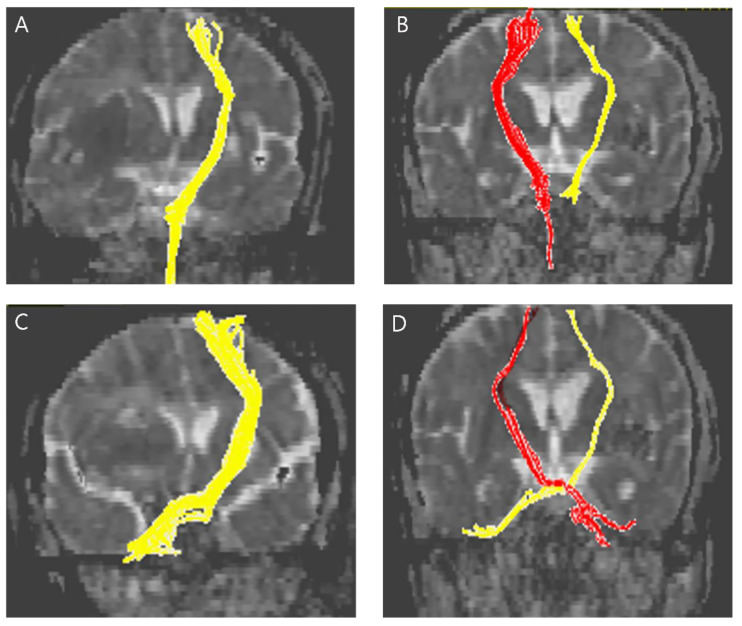
Representative diffusion tensor tractography images of the CST in typical subjects from the (**A**) poor hand function group and the (**B**) good hand function group. The non-affected tract is shown in yellow in (**A**) and red in (**B**). Representative diffusion tensor tractography images of the CPCT in typical subjects from the (**C**) poor hand function group and the (**D**) good hand function group. The non-affected tract is shown in yellow in (**C**) and red in (**D**).

The normalized FA and FN values in the CPCT were significantly higher in the good hand function group than in the poor hand function group (FN 25.240 and 0.587 respectively, *p* = 0.021; FA 0.985 and 0.459 respectively; *p* = 0.015) (Figure 2). The normalized FN and FA in the CST were not significantly different between the poor hand function group and the good hand function group. Representative DTIs of the CST and CPCT for the two groups are presented in Figure 3.

**Figure 4:**
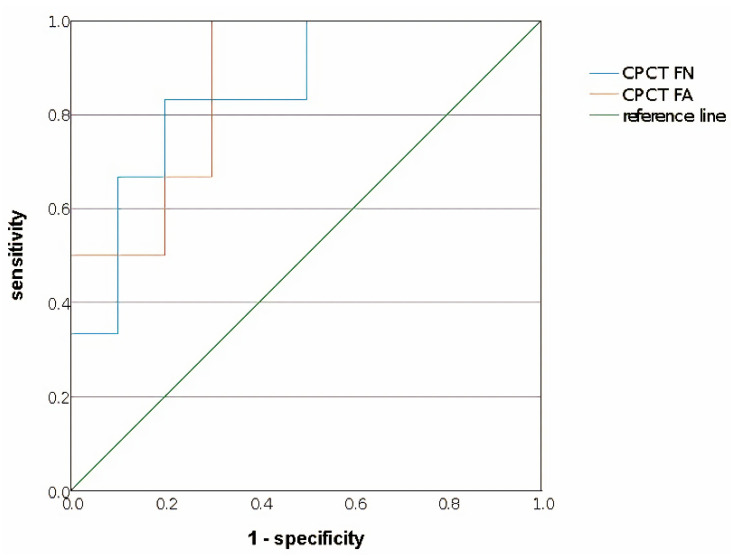
Significant variables for cut-off values that can distinguish the good hand function group from the poor hand function group in the ROC curve analysis. The normalized (affected/non-affected) cut-off value of the highest accuracy (0.867) was 0.8889 for the FA in the CPCT. The sensitivity was 0.833 and the specificity was 0.7. Blue line, normalized CPCT FN; orange line, normalized CPCT FA; green line, reference line.

Based on the ROC curve, values that could be cut-off values with an area between 0.8–0.9 which means a good level of accuracy to distinguish the good hand function group from the poor hand function group in stroke patients included the FN and FA in the CPCT (Table 3). Accuracy for the normalized FN in the CPCT was 0.850 and normalized FA in the CPCT was 0.867. Between them, we decided to find the cut-off value with the higher accuracy, which was the normalized FA in the CPCT. The cut-off value for a sensitivity of 0.833 and specificity of 0.7 was found to be 0.8889 (Figure 4, Supplementary Table S1).

## 4. Discussion

When designing this study, we attempted to find the relevance of the CST and the cerebellar afferent tract with regard to hand function. As described above, there are many patients who have difficulties in daily life due to the deterioration of hand function in the chronic period after a stroke [1,2], and hand function has a low incidence of recovery beyond six months after the onset of a stroke [22]. Therefore, in this study, we hypothesized that the status of these white matter tracts in the acute phase might be related to hand function in the chronic phase. As expected, in the CPCT the FA and FN values in the acute phase were significantly lower in the poor hand function group than in the good hand function group based on the hand function test conducted in the chronic phase. Furthermore, it was possible to present an acute phase cut-off value that could predict good hand function in the chronic phase after a stroke. However, the FN and FA values in the CST in the acute phase did not show a significant relationship with hand function in the chronic phase.

Our main findings demonstrate that FA and FN values in the CPCT at the acute stage after a stroke are related to chronic hand function, and this is in accordance with several previous studies. Damage to the frontal/parietal cortex and the territory of the deep middle cerebral artery, including the internal capsule and lentiform nucleus, affects the cerebellum through diaschisis [10,18,23,24]. The affected cerebrum causes hypoperfusion or hypometabolism of the contralateral cerebellum [21,22] and it can also affect the CPCT [10,25,26,27]. As such, the cerebellum affected by supratentorial stroke may have changes in its neuronal activities and connections [26], through hypoperfusion or hypometabolism due to diaschisis. Patients with more than one-third of the cerebral hemisphere affected were found to have decreased FA values in the contralateral middle cerebellar peduncle [28]. Functional imaging has revealed that cerebellar activity after a stroke contributes to sensorimotor control [29], training gains [30], and spontaneous motor recovery [31]. In a recent study, it was demonstrated that chronic CPCT integrity is related to fine hand motions independent of the CST [20]. Through this, it can be seen that the cerebral hemispheres can be activated when the cerebellum is stimulated. For example, when the cerebellum is stimulated with low-frequency electrical stimulation, the primary motor cortical excitatory circuit is activated [32,33]. It might be a useful addition for future treatment strategy in clinical stroke care.

In this study, it was found that the FN and FA values in the CST in the acute phase were not associated with hand function in the chronic phase. There are controversial ideas on the relationship between CST integrity and hand function. Some studies have reported that there is a significant relationship between FA values in the CST and hand function [3,34], but there is also research that shows that this is not the case. Representatively, in the study of Buetefisch et al. [35], there was no significant relationship between FA values in the CST and hand function in patients with chronic ischemic stroke. The authors explained that, firstly, FA values in the CST were measured not only in the M1 but in the entire CST [36], and secondly, the study included patients whose motor-evoked potential was measured, which means that the CST was relatively preserved [35]. In the present study, we speculate that there was no significant association between FA values in the CST in the acute phase and hand function in the chronic phase for the following two reasons. First, FA values in the mature CST in the chronic phase will eventually be related to hand function. This can be indirectly affirmed through Yoo et al.’s study [3] that found that FA values in the CST were related to hand function beyond six months after a stroke. Second, the concept of hand function includes not only power but also dexterity. To accurately measure hand function it is necessary to conduct a test that can measure dexterity, as in this study, rather than simply including power [37]. Although it is not a hand function, functional indicators such as the Berg Balance Scale (BBS) and the Functional Ambulation Categories (FAC) measured six months after a stroke were not related to acute phase FA values in the CST [9].

Our study has a few limitations. First, because our study was a retrospective longitudinal observational study, the sample size was relatively small. If the sample size was slightly larger, statistical analyses such as linear regression would be possible, which would have been helpful to determine a more accurate cut-off value. And the effect of the white matter tract on each item of the Jebson’s Hand Function test could be examined. Also, hemorrhagic stroke patients comprised 18.2% of the poor hand function group and 42.9% in the good hand function group, although it is not statistically different. (*p* = 0.326) The distribution of stroke type could be rather equal if the sample size was slightly larger. Second, if hand function was investigated with various items such as grip strength in addition to the Jebson’s Hand Function Test, the effect of power and dexterity could be determined thoroughly. However, in previous studies, it was difficult to examine the effect of dexterity by measuring hand function only with grip strength, and we tried to overcome this limitation [38]. Third, if DTI and the hand function test had been done during both the acute and chronic phases after a stroke, it would have provided a more detailed look at longitudinal relationships according to hand function.

In conclusion, the white matter integrity of the cerebellar afferent pathway, representative as FA and FN values of CPCT, within two months after stroke onset might reflect the hand function at the fully recovered chronic stage in patients with stroke. We present that the normalized cut-off value of CPCT as 0.8889 within two months after onset may predict good hand function at the chronic stage in patients with stroke. These results would be clinically useful for prediction of prognosis and making treatment plans in patients with stroke.

## Figures and Tables

**Table 1:** The participants’ demographic data.

	Poor Hand Function Group (*n* = 11)	Good Hand Function Group (*n* = 7)	*p*
Age, years	56.0 (50.2–63.1)	62.1 (46.6–67.3)	0.389
Sex			0.335
Female, *n* (%)	7 (63.6)	2 (28.6)	
Male, *n* (%)	4 (36.4)	5 (71.4)	
Stroke type			0.326
Hemorrhage, *n* (%)	2 (18.2)	3 (42.9)	
Infarct, *n* (%)	9 (81.8)	4 (57.1)	
Brain injury location			0.434
Cortex, *n* (%)	4 (36.4)	3 (42.9)	
Subcortex, *n* (%)	1 (9.1)	2 (18.2)	
Mixed, *n* (%)	6 (54.5)	2 (18.2)	
Hemispheric brain lesion			1.000
Left, *n* (%)	7 (63.6)	4 (57.1)	
Right, *n* (%)	4 (36.4)	3 (42.9)	

Values are the median (interquartile range: first–third quartiles) or number (*n*) (%). *p*-values were tested using Pearson’s chi-square test for age and brain injury location, and Fisher’s exact test for sex, stroke type, and hemispheric lesion.

**Table 2:** FN and FA values in the CST and CPCT by group.

	Values	Poor Hand Function Group	Good Hand Function Group	*p*
CST	FN	0.206 (0.019–0.453)	0.492 (0.168–0.827)	0.327
	FA	0.475 (0.187–0.764)	0.823 (0.480–1.017)	0.176
CPCT	FN	0.587 (0.005–1.696)	25.240 (0.548–62.006)	0.021
	FA	0.459 (0.176–0.742)	0.985 (0.922–1.041)	0.015

Values are the mean (confidence interval), and these are FN and FA in the poor hand function group and good hand function group normalized as affected/non-affected. CST, corticospinal tract; CPCT, cortico-ponto-cerebellar tract; FN, fiber number; FA, fractional anisotropy. Comparisons between the poor hand function group and good hand function group were calculated with the Mann-Whitney U test with Bonferroni correction (*p* < 0.025 is considered to be significant).

**Table 3:** ROC curve analyses.

	Values	AUC	SE	*p*-Value
CST	FN	0.708	0.139	0.175
	FA	0.775	0.131	0.074
CPCT	FN	0.850	0.100	0.023
	FA	0.867	0.091	0.017

Values for FN and FA were normalized as affected/non-affected. ROC, receiver operating characteristic; AUC, area under the curve; SE, standard error; CST, corticospinal tract; CPCT, cortico-ponto-cerebellar tract; FN, fiber number; FA, fractional anisotropy. Comparisons between the poor hand function group and good hand function group were calculated by ROC curve analyses (*p* ≤ 0.05 is considered to be significant).

## Data Availability

The data presented in this study are available on request from the corresponding author.

## Supplementary Materials

The following supporting information can be downloaded at: https://www.mdpi.com/article/10.3390/life12070959/s1, Table S1: Sensitivity and specificity when each value of CPCT FA becomes a cut-off value.
