# Supplementary material for: Preservation of Cerebellar Afferent Pathway May Be Related to Good Hand Function in Patients with Stroke
Source: Life (Basel). 2022 Jun 26;12(7):959. doi: 10.3390/life12070959 (PMC9318318; doi:10.3390/life12070959)
Supplement: Supplementary file 1 [file life-12-00959-s001.zip › life-1762138-supplementary.pdf]

Supplementary Table S1. Sensitivity and specificity when each value of CPCT FA becomes a cut-off value.

	Values	Sensitivity	Specificity
CPCT FA	0.413	1.000	0.5
	0.829	1.000	0.6
	0.849	1.000	0.7
	0.889	0.833	0.7
	0.922	0.667	0.7
	0.947	0.667	0.8
	0.967	0.500	0.8
	0.999	0.500	0.9
	1.038	0.500	1.0
	1.053	0.333	1.0
	1.060	0.167	1.0
	2.062	0.000	1.0

Values were normalized as affected/non-affected. The optimum normalized cut-off value of CPCT FA was 0.889 with the sensitivity of 0.833, and the specificity of 0.7.

CPCT, cortico-ponto-cerebellar tract; FA, fractional anisotropy.
